# Identification of molecular subgroups in osteomyelitis induced by *staphylococcus aureus* infection through gene expression profiles

**DOI:** 10.1186/s12920-023-01568-x

**Published:** 2023-06-27

**Authors:** Xiangwen Shi, Haonan Ni, Linmeng Tang, Mingjun Li, Yipeng Wu, Yongqing Xu

**Affiliations:** 1 Kunming Medical University, Kunming, China 650500; 2 Laboratory of Yunnan Traumatology and Orthopedics Clinical Medical Center, Yunnan Orthopedics and Sports Rehabilitation Clinical Medical Research Center, Department of Orthopedic Surgery, 920th Hospital of Joint Logistics Support Force of PLA, Kunming, Yunnan P.R. China 650100; 3 Bone and Joint Imaging Center, Department of Medical imaging, The First Affiliated Hospital of Hebei North University, Zhangjiakou, China 075000

**Keywords:** Osteomyelitis, *Staphylococcus aureus*, Molecular subgroups, Bioinformatics

## Abstract

**Background:**

*Staphylococcus aureus* (*S. aureus*) infection-induced osteomyelitis (OM) is an inflammatory bone disease accompanied by persistent bone destruction, and the treatment is challenging because of its tendency to recur. Present study was aimed to explore the molecular subgroups of *S. aureus* infection-induced OM and to deepen the mechanistic understanding for molecularly targeted treatment of OM.

**Methods:**

Integration of 164 OM samples and 60 healthy samples from three datasets of the Gene Expression Omnibus (GEO) database. OM patients were classified into different molecular subgroups based on unsupervised algorithms and correlations of clinical characteristics between subgroups were analyzed. Next, The CIBERSORT algorithm was used to evaluate the proportion of immune cell infiltration in different OM subgroups. Weighted gene co-expression analysis (WGCNA) was used to identify different gene modules and explore the relationship with clinical characteristics, and further annotated OM subgroups and gene modules by the Gene Ontology (GO) and Kyoto Encyclopedia of Genes and Genomes (KEGG) analysis.

**Results:**

Two subgroups with excellent consistency were identified in this study, subgroup and hospital length of stay were independent predictors of OM. Compared with subgroup I, OM patients in subgroup II had longer hospital length of stay and more severe disease. Meanwhile, the infiltration proportions of monocytes and macrophages M0 were higher in patients of OM subgroup II. Finally, combined with the characteristics of the KEGG enrichment modules, the expression of osteoclast differentiation-related genes such as CTSK was upregulated in OM subgroup II, which may be closely associated with more severe OM patients.

**Conclusion:**

The current study showed that OM subgroup II had longer hospital length of stay and more severe disease, the osteoclast differentiation pathway and the main target CTSK contribute to our deeper understanding for the molecular mechanisms associated with *S. aureus* infection-induced OM, and the construction of molecular subgroups suggested the necessity for different subgroups of patients to receive individualized treatment.

**Supplementary Information:**

The online version contains supplementary material available at 10.1186/s12920-023-01568-x.

## Introduction

Osteomyelitis (OM) is an inflammatory process of continuous bone destruction caused by bacteria, fungi, or other pyogenic organisms. Long treatment time and uncontrollable recurrence make it one of the refractory infections in the field of orthopedics [1, 2]. It can generally infect patients of any age, while diabetes, peripheral vascular disease, and immune deficiencies make patients more susceptible to OM [3]. As far as the route of infection is concerned, OM mostly comes from hematogenous or persistent exogenous infection, and can invade almost any part of the bone, including bone marrow, bone cortex and even peripheral soft tissue [4]. The number of OM patients in the United States had doubled in the past 41 years, and the cost of repeated treatment per OM patient can be as high as $600,000 [5, 6]. In addition to the heavy economic burden, the frequent recurrence of OM has led to a mortality rate of nearly 8%, which is even higher in developing countries [7] and this is incredible data.

OM often involves complex infections of multiple strains, so the treatment is very tricky. Generally speaking, the treatment of OM requires the implantation of antibiotic-loaded fillers after complete surgical removal of dead bone, as well as the continuous administration of sufficient concentrations of antibiotics [8]. However, more than 75% of OM infections are caused by the opportunistic Gram-positive *Staphylococcus aureus* (*S. aureus*) [9]. It readily forms a dense biofilm of DNA and proteins, which is considered to be a barrier to treatment of OM. Specifically, the formation of biofilm will prevent antibiotics from entering the site of action, which results in bacteria becoming resistant to antimicrobials and decreasing the success rate of treatment [10, 11]. On the other hand, with increasing microbial drug resistance and imperfect properties of implanted materials, the traditional treatment still struggles to improve the cure rate. Although researchers devote substantial time and effort to studying the potential therapeutic mechanism of OM, the exact mechanism of occurrence and development remains unknown. Previous studies demonstrated that *S. aureus* can inhibit osteoblast differentiation and promote the production of RANKL, which stimulated osteoclast differentiation and growth [12, 13]. Other studies found that the development of *S. aureus* infection-induced OM involved MAPK and Wnt signaling pathways of, accompanied by an increase in osteoclast numbers [14]. However, due to the lack of in-depth research on the mechanism, the molecular pathophysiological process of OM remains a difficult problem.

**Fig. 1:**
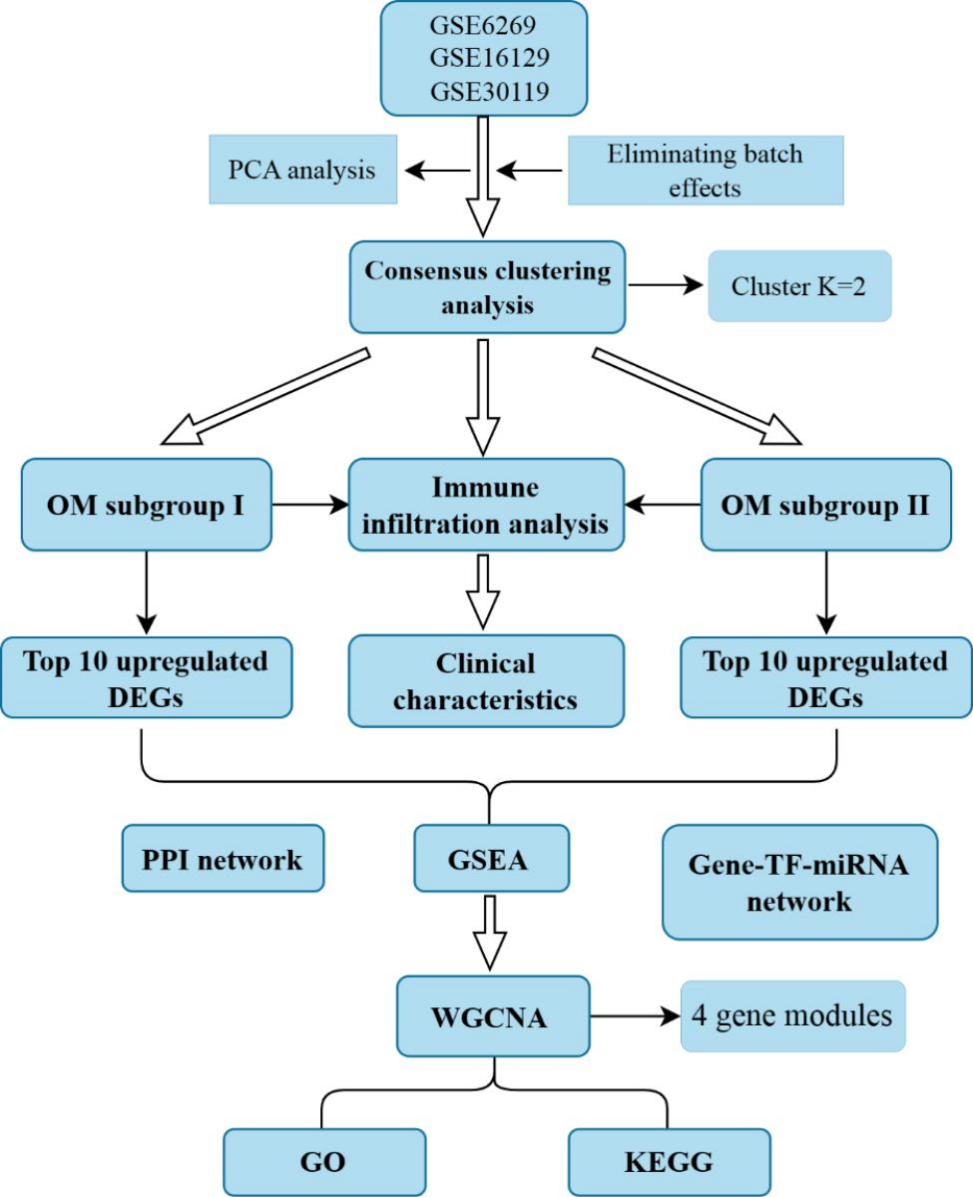
Flow chart of the study

With the continuous development of bioinformatics and high-throughput sequencing technology have led to an increasing number of studies using bioinformatics to examine the specific occurrence and development mechanisms of OM. Chen et al. [15] analyzed the differentially expressed genes (DGEs) in OM caused by *S. aureus* by using public database chips, and further revealed the biological function and pathway mechanism of differential genes. However, most of the previous studies focused on the DGEs between OM samples and normal samples, there were comparatively few in-depth studies on DGEs between OM samples. To better understand the pathogenesis of OM caused by *S. aureus*, we divided OM patients into different molecular subgroups, established a gene co-expression module, and discussed the clinical relationship and deep molecular mechanism of each subgroup (Fig. 1).

## Materials and methods

### Collection and annotation of GEO microarray data

Three gene expression datasets (GSE6269, GSE16129 and GSE30119) of OM and healthy samples were downloaded and obtained for free through The Gene Expression Omnibus (GEO) website (https://www.ncbi.nlm.nih.gov/geo/). GSE6269 and GSE16129 came from GPL96, while GSE30119 was based on GPL6947. The extracted information included probe matrix files and gene matrix files. We translated the probe names into gene names using the programming language Perl (http://www.perl.org/) [16], then used the “limma” and “sva” packages [17] of R software to merge the three gene expression datasets to obtain the gene expression profiles of 164 OM patients and 60 healthy controls. Log2 was used to convert large numerical data. As the merged microarray data come from three different gene chips, we applied the “ComBat” method to correct the data in batches to eliminate the batch effects caused by multiple factors [18]. Finally, the principal component analysis was performed with the R/ggplot2 package to determine whether batch effects was removed.

### Construction of subgroups based on consensus clustering

The consensus clustering uses quantitative and visual methods to obtain a centralized estimate of data, building a more meaningful subgroup. The consensus clustering was performed using the “limma” and “ConsensusClusterPlus” packages of R/Bioconductor package [19], and specifically, the homogenized gene matrix was passed to the consensus clustering algorithm to obtain each cluster membership of OM sample. The maximum number of clusters was set at 10 and the final number of clusters was determined by the concensus index, cumulative distribution function (CDF) and cluster consistency score > 0.75.

### Immune infiltration analysis

The CIBERSORT algorithm [20] was used to calculate the infiltration abundance of 22 immune cell types in each OM and healthy sample to explore the differences in immune microenvironment between OM and healthy patients. Based on the different clustering results, the abundance of immune cell infiltration between different OM subgroups was explored.

### Comparison of clinical characteristics of different subgroups

From the previously downloaded probe matrix files of the three gene datasets, we extracted relevant clinical characteristics, such as gender, age, race, pathogen species, OM type, severity, and hospital length of stay, and compared the two subgroups for clinical differences. Categorical variables such as gender, race, pathogen species, severity, and OM type, were compared using pairwise data comparison to create a histogram. Continuous variables such as age and hospital length of stay, were compared using the Wilcoxon’s rank-sum test to create a boxplot.

### Identification of upregulated genes in subgroups

In each subgroup, the “limma” package in R software was used to identify genes that were upregulated in comparison with the control group. The threshold of mean difference is greater than 0.2, and the adjusted *P* value is less than 0.05.

### Protein-protein interaction (PPI) and gene-TF-miRNA interaction networks

The PPI network of top 10 upregulated DEGs was constructed through the STRING online website (https://cn.string-db.org/). To show the interactions of the 10 DEGs as much as possible, the minimum required interaction score was set to: low confidence (0.15). The gene-TF-miRNA interaction network was further constructed by miRTarBase v8.0 and ENCODE ChIP-seq data in NetworkAnalyst online website (https://www.networkanalyst.ca/).

### Gene Set Enrichment Analysis (GSEA) of subgroup

The comparison files of each subgroup and the control group were converted into gene list files and gene dataset files by Perl software, and then the files of the two subgroups were divided into two times for GSEA analysis by GSEA software (version 4.3.2) [21]. The run options were set to a minimum of 15 and a maximum of 5000 so that data criteria for larger gene sets can be met. In the results, *P* values and false discovery rate (FDR) less than 0.05 were considered significantly enriched.

### Construction of weighted gene co-expression Network Analysis (WGCNA)

A weighted co-expression network was constructed by the WGCNA package in R software to determine the biological function of specific genes in each subgroup that are representative [22]. Initially, based on the scale-free network model, a “pickSoftThreshold” function is used to find the most appropriate soft threshold that balances the scale independence and average connectivity of the co-expression network. Next, the topological overlap matrix (TOM) and its corresponding (1-TOM) values were calculated, and they were used as distance metrics to merge the highly correlated modules. Finally, the correlation coefficients and *P* values between each expression module and clinical features were calculated using the Pearson correlation analysis and visualized by a heatmap.

### Gene Ontology (GO) and pathway Enrichment Analysis

The GO database and Kyoto Encyclopedia of Genes and Genomes (KEGG) Pathway Enrichment database (https://www.kegg.jp/kegg/pathway.html) [23] are important databases for the analysis of gene biological processes and molecular functions, facilitating our search for clinical therapeutic directions at the mechanistic level. Enrichment results for the four gene modules were derived using the “enrichplot” package and the “clusterProfiler” package and visualized as bubble plots [24]. It is worth noting that we set the *P* value filter condition to 0.05 and the corrected *P* value filter condition to 1. To further understand the pathway mechanism, the most significantly upregulated genes from each module were screened to show the detailed gene enrichment of each subgroup in the four pathways and visualized by a heatmap.

## Results

### Characteristics of OM sample

All study samples were obtained from three separate GEO genetic datasets. The GSE6269 dataset included 19 OM samples and 6 healthy controls, the GSE16129 dataset included 46 OM samples and 10 healthy controls, and the GSE30119 dataset included 99 OM samples and 44 healthy controls. All three databases provide information on patient gender, age, and ethnicity. The remaining clinical information including pathogen species, OM type, severity, and total hospital stay was obtained from the GSE30119 dataset.

### Evaluation of eliminating batch effects

**Fig. 2:**
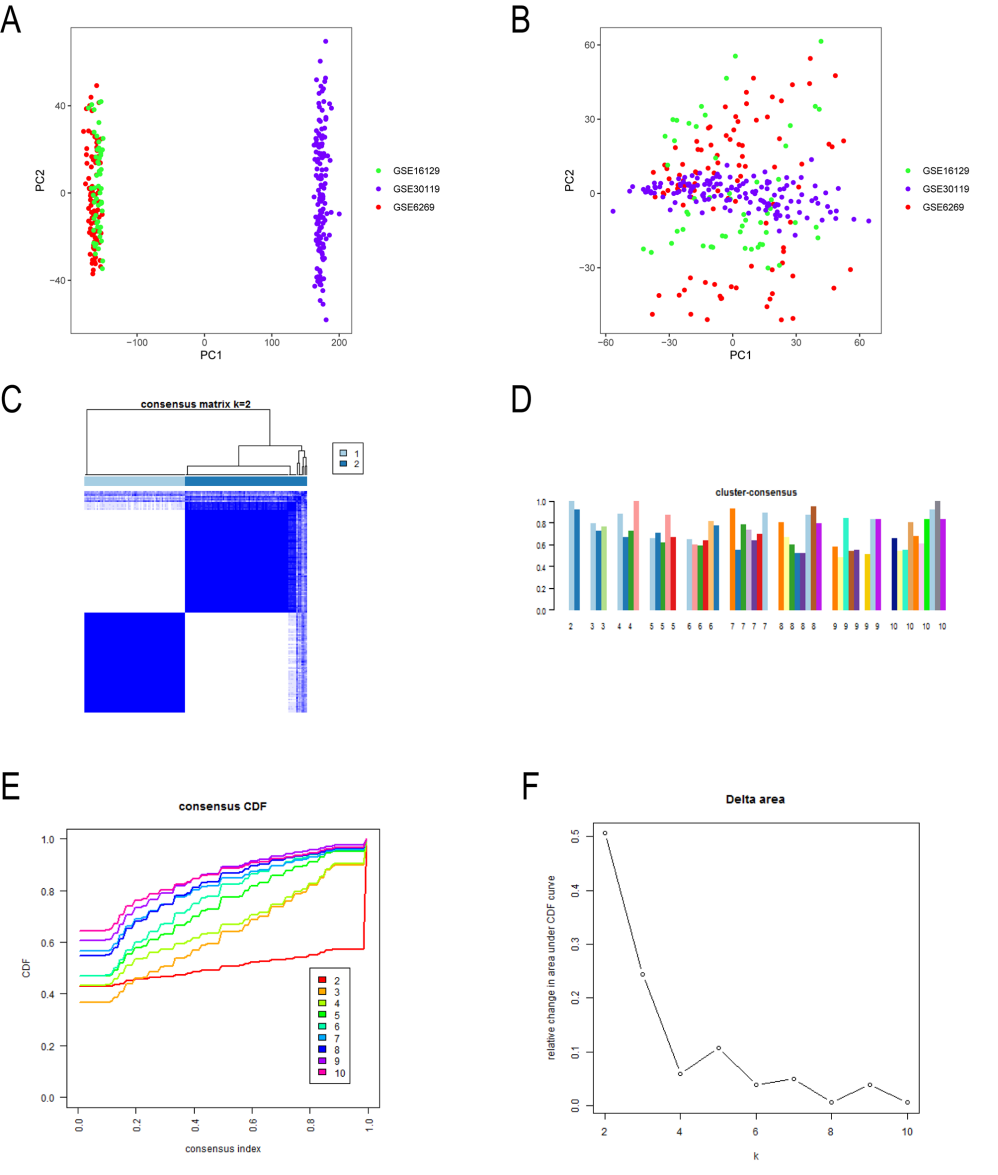
Principal component analysis (PCA) and consensus clustering. A Before eliminating the batch effect. B Elimination of batch effect through PCA, data from the three datasets were evenly distributed in the same region. C Gene expression clustering map of two subgroups. The deeper blue rectangle in the subgroup, the stronger the genetic correlation. D Nine classification methods are obtained by consensus clustering analysis. The abscissa represents different groups, and the ordinate indicates consensus clustering score. E Cumulative distribution graph of nine classification methods. F Delta area of nine classification methods

The principal component analysis (PCA) cluster diagram was used to evaluate the batch effect between GSE6269, GSE16129 and GSE30119 datasets. Before eliminating the batch effect, the data of the three gene datasets are concentrated in two different parts (Fig. 2A). To eliminate the batch effect, the “sva” package in R was used to normalize the above data. After standardized processing, data from the three gene datasets were evenly distributed in the same region, indicating that the batch effect was successfully eliminated (Fig. 2B).

### Consensus clustering analysis of OM sample

To explore the relationship between subgroups and clinical characteristics, consensus clustering analysis was performed on the corrected gene expression files and all 164 OM samples. After setting the maximum value of the clustering variable (K) to 10, a total of 9 clusters were obtained (Fig. 2C). When the clustering variable was 2, all OM samples were divided into two subgroups. The clustering score of each subgroup was greater than 0.75 (Fig. 2D), indicating that the two subgroups had high agreement and similarity. Two subgroups were finally confirmed by combining the results of concensus index and CDF curves (Fig. 2E F). The top 10 upregulated DEGs were shown in Table 1.

**Table 1:** Top 10 specifically upregulated genes in the two subgroups

Subgroup	DEGs						
Subgroup I	PRKCQ/FAIM3/CD2/CD6/BCL11B/SPOCK2/PRKCH/AES/PPP3CC/SKAP1						
Subgroup II	DYSF/CCPG1/GYG1/F5/KIF1B/PYGL/CDA/PGD/BST1/GCA						

Abbreviations: DEGs: differentially expressed genes.

**Table 2:** Analysis of variance for subgroups and age

	Degree of freedom	Sum square	Mean square	F value	*P* value
Subgroup	1	133.9	590.2	3.245	0.0434*
Subgroup: Age	1	1961	227.3	1.25	0.2914
Residuals	159	1641.5	181.9		

Abbreviations: *: *P* < 0.05; **: *P* < 0.01; ***: *P* < 0.001.

### Immune microenvironment analysis in OM patients

**Fig. 3:**
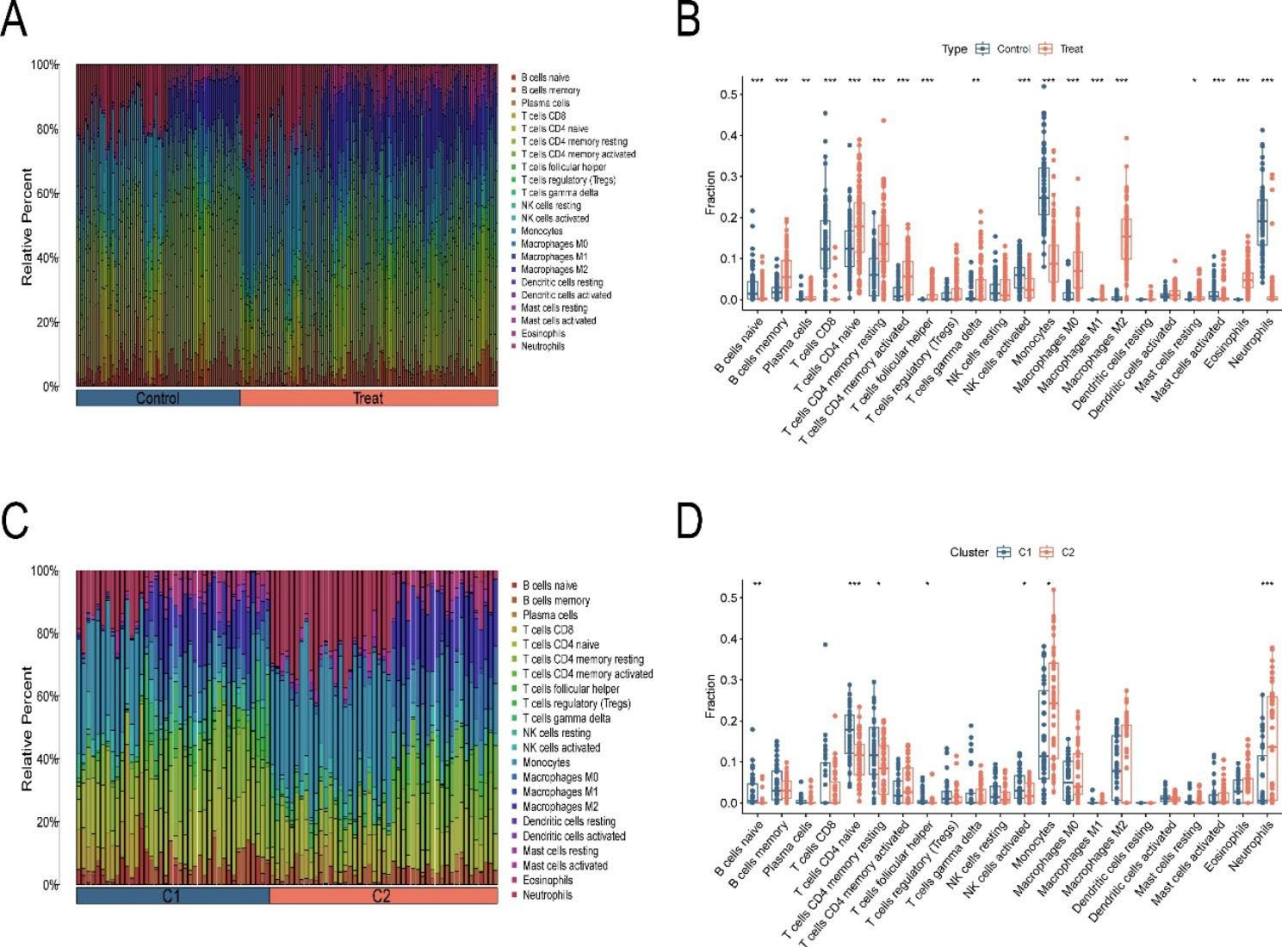
Immune microenvironment in OM patients and different OM subgroups. A and B Differences in the proportion of 22 immune cell infiltrations between OM patients and healthy controls. C and D Differences in the proportion of 22 immune cell infiltrations between OM subgroup I and subgroup II. *P* values were showed as: *: *P* < 0.05; **: *P* < 0.01; ***: *P* < 0.001

Based on the CIBERSORT algorithm we further observed the immune microenvironment in 164 OM patients (Fig. 3A and B), and the results suggested that 18 of 22 immune cells were involved in the regulation of the immune microenvironment in OM, of which 12 immune cells had significantly higher infiltration abundance in OM patients and 6 immune cells had lower infiltration abundance in OM patients. Moreover, we further explored the differences in the immune microenvironment of two different OM subgroups (Fig. 3 C and 3D), suggesting that monocytes and macrophages M0 were significantly highly expressed in subgroup II.

### Comparison of clinical information among subgroups

**Fig. 4:**
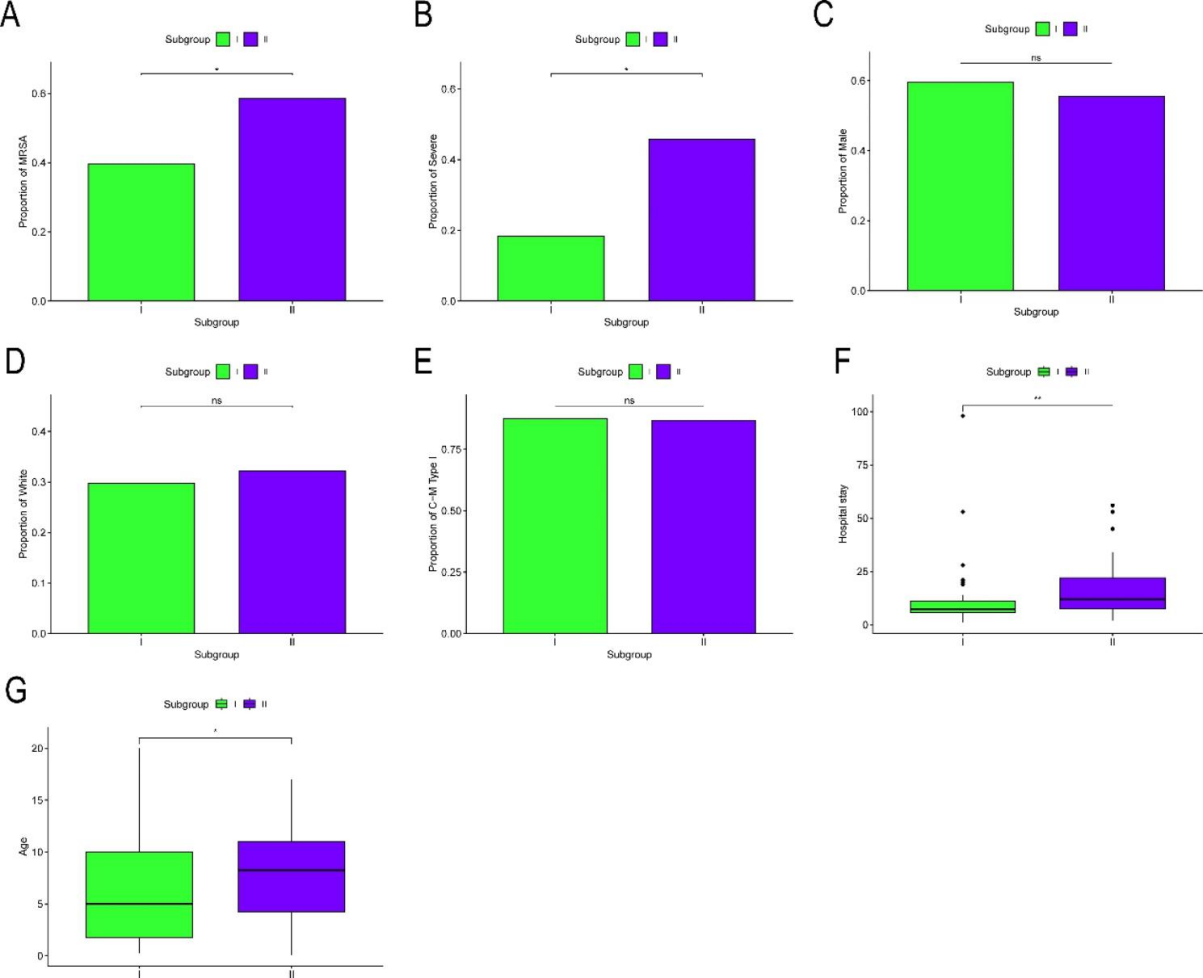
Analysis of differences in clinical characteristics between two subgroups. A Histogram of the proportion of MRSA between two subgroups. B Histogram of the proportion of severe between two subgroups. C-E Histogram of differences of gender, race, and OM classification between two subgroups. F Box plot of differences of hospital stay between two subgroups. G Box plot of age differences between two subgroups. *P* values were showed as: ns: *P* > 0.05; *: *P* < 0.05; **: *P* < 0.01; ***: *P* < 0.001

The clinical characteristics were extracted from probe matrix file, and the differences of information between the above two subgroups were compared. For categorical variables, the results of pathogen species comparison suggested that the number of pathogenic bacteria as MRSA was higher in subgroup II than in subgroup I (*P* < 0.05) (Fig. 4A). The results of OM severity grading indicated that the severity was higher in subgroup II than in subgroup I (*P* < 0.05) (Fig. 4B). However, there were no significant differences in the remaining categorical variables such as sex, race, and OM classification among the two subgroups (*P* > 0.05) (Fig. 4 C-4E).

For continuous variables, the results of comparing hospital length of stay indicated that the hospital length of stay was longer in subgroup II than in subgroup I (*P* < 0.001) (Fig. 4F). Besides, there was no significant difference in the age of the patients among the two subgroups (*P* > 0.05) (Fig. 4G).

### PPI and gene-TF-miRNA networks

**Fig. 5:**
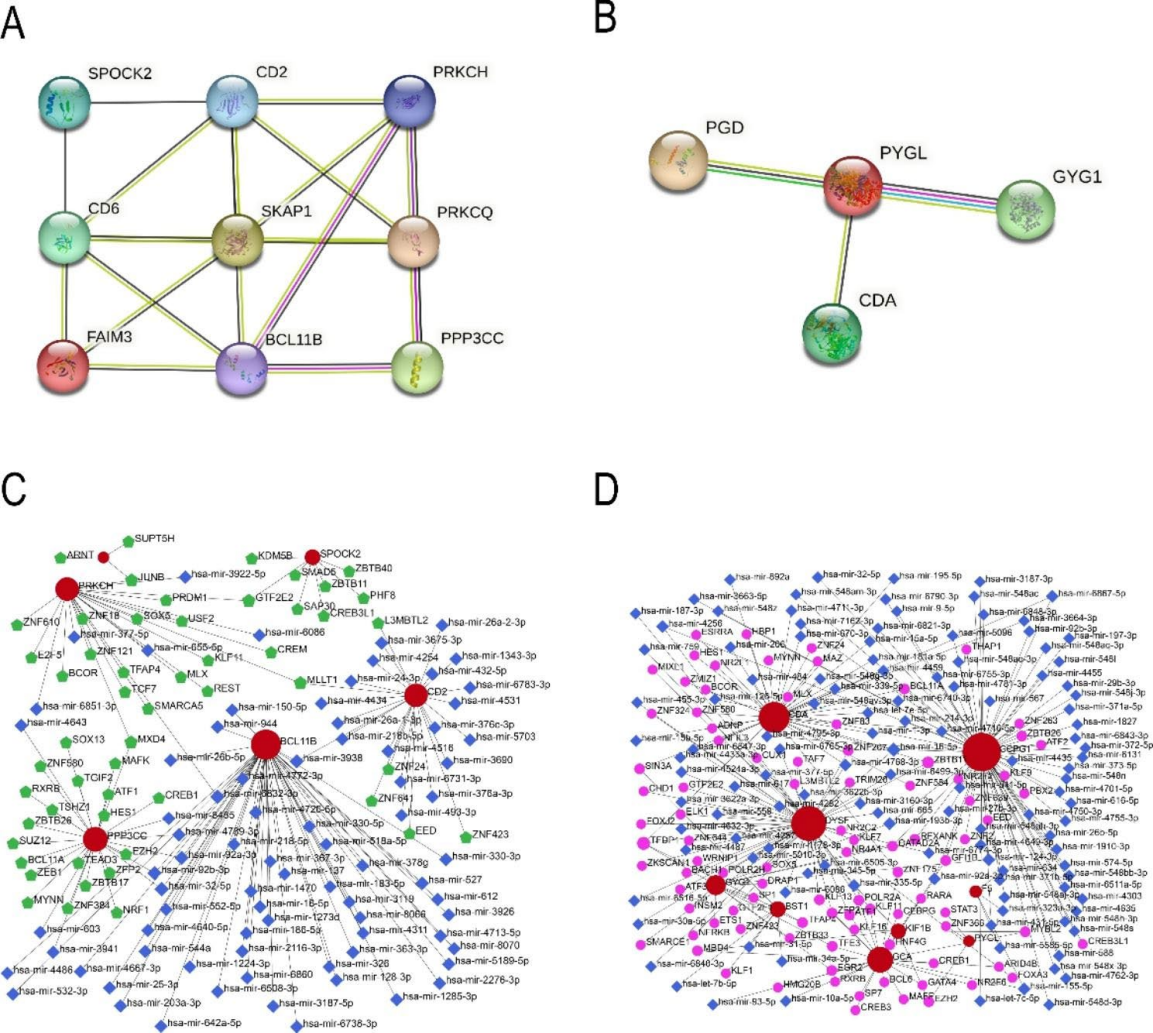
Protein-protein interaction (PPI) and gene-TF-miRNA networks. A PPI network based on the top 10 upregulated genes in subgroup I, including 9 nodes and 17 edges. B PPI network based on the top 10 upregulated genes in subgroup II, including 4 nodes and 3 edges. C Gene-TF-miRNA network based on the top 10 upregulated genes in subgroup I, including 138 nodes and 141 edges. D Gene-TF-miRNA network based on the top 10 upregulated gene in subgroup II, including 225 nodes and 251 edges. Nodes without interactions were removed

To explore the underlying molecular mechanisms of top 10 upregulated DEGs in subgroup I and subgroup II, PPI networks were further constructed, with a total of 9 nodes and 17 edges in subgroup I network and 4 nodes and 3 edges in subgroup II network (Fig. 5A and B). Additionally, the gene-TF-miRNA network in subgroup I included 138 nodes and 141 edges and the gene-TF-miRNA network in subgroup II included 225 nodes and 251 edges (Fig. 5 C and 5D).

### GSEA analysis of different OM subgroups

**Fig. 6:**
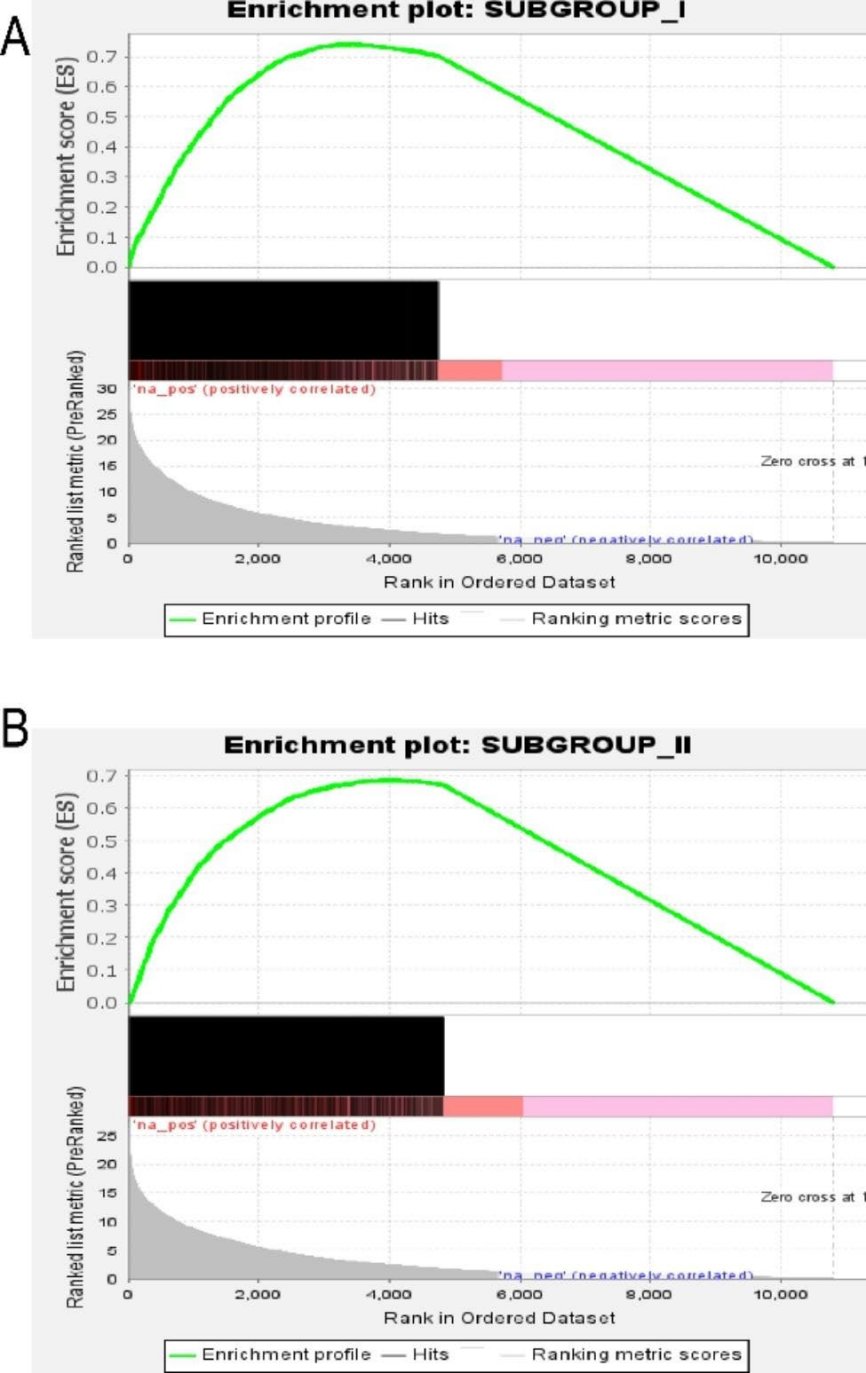
GSEA analysis of the subgroups. A GSEA analysis of subgroup (I) B GSEA analysis of subgroup (II) The green curve represents the enrichment score (ES) of the subgroup gene set. The black vertical line is the position of the unique DGEs in the gene ranking list. The gray area reflects the signal-to-noise ratio between each subgroup and the control group

Through pairwise comparison between each subgroup, 2127, 1907 and 65 upregulated DEGs were found in subgroup I and subgroup II, respectively (the threshold of mean difference > 0.2 and the adjusted *P* < 0.05) (Additional file 1). The highest point of the green curves represents the enrichment score (ES) of the subgroup gene set, the black lines represent the unique DGEs in the subgroup, and the gray lines represent the signal-to-noise ratio between the subgroup and the healthy sample. The black and gray areas of the two subgroups were all concentrated on the left side of the image, and their FDR and *P* values were all less than 0.001, suggesting that the specific DGEs among different subgroups and between the subgroups and healthy samples were consistent (Fig. 6A and B). Notably, the clinical severity and hospital length of stay were also greater in subgroup II than in subgroup I, which might suggest that patients in subgroup II were more severe.

### WGCNA analysis and clinical characteristics

**Fig. 7:**
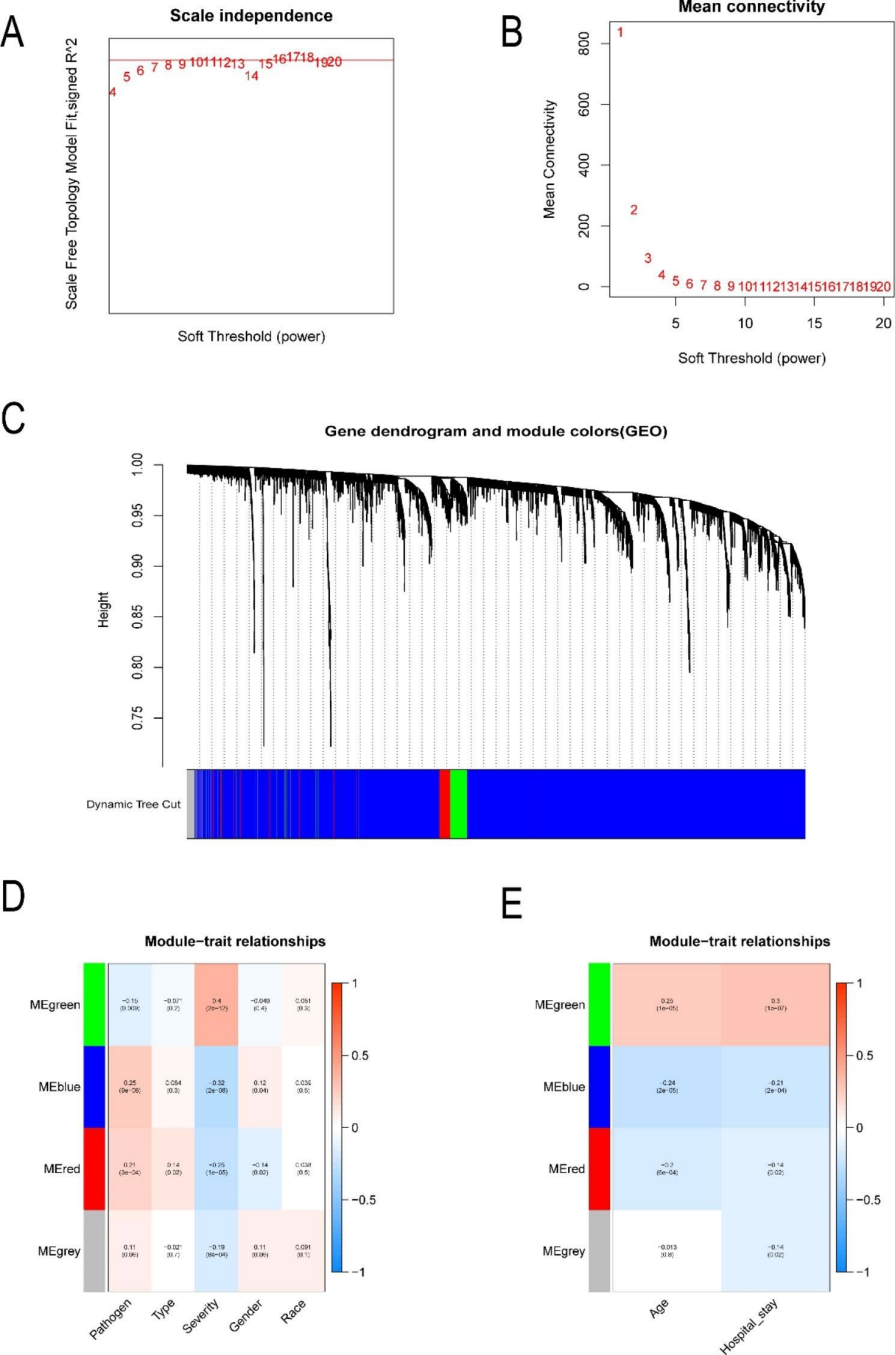
WGCNA analysis of the two subgroups. A and B The association of various soft-thresholding powers with the scale-free fitting Index and the mean connectivity. C Clustering dendrogram of genes. D The relationships between genes of each module and categorical variables such as pathogen, type, severity, gender, and race. E The relationships between genes of each module and continuous variables such as age and hospital length of stay

After determining the soft threshold of 6 (Fig. 7A and B), a total of 4145 up-regulated specific DGEs were incorporated into WGCNA analysis. Next, four modules are removed from the identification of gene clustering tree species (Fig. 7C). There are 3830 genes in the blue module, 134 genes in the green module, 110 genes in the red module and 69 genes in the grey module. We summarized the top 50 genes with the most significant differences in each gene module (Additional file 2).

In addition, the association between each module and the clinical characteristics of OM patients was demonstrated through graphical visualization. For categorical variables, genes in the blue, red, and gray modules were negatively associated with more severe OM patients, while the green module showed a synergistic relationship. Genes in the blue and red modules were positively associated with MRSA-infected OM, while the green module was negatively associated. Male OM patients had more expressed genes in the blue module, and conversely, the red module showed a slight negative correlation. In addition, Cierny-Mader type I patients expressed more genes in the red module (Fig. 7D). For continuous variables, blue and red modules were negatively correlated with age and hospital length of stay of OM patients, while green was positively correlated with these two indictors. The gray module was only negatively correlated with the total length of stay of OM patients and had no correlation with age (Fig. 7E).

**Fig. 8:**
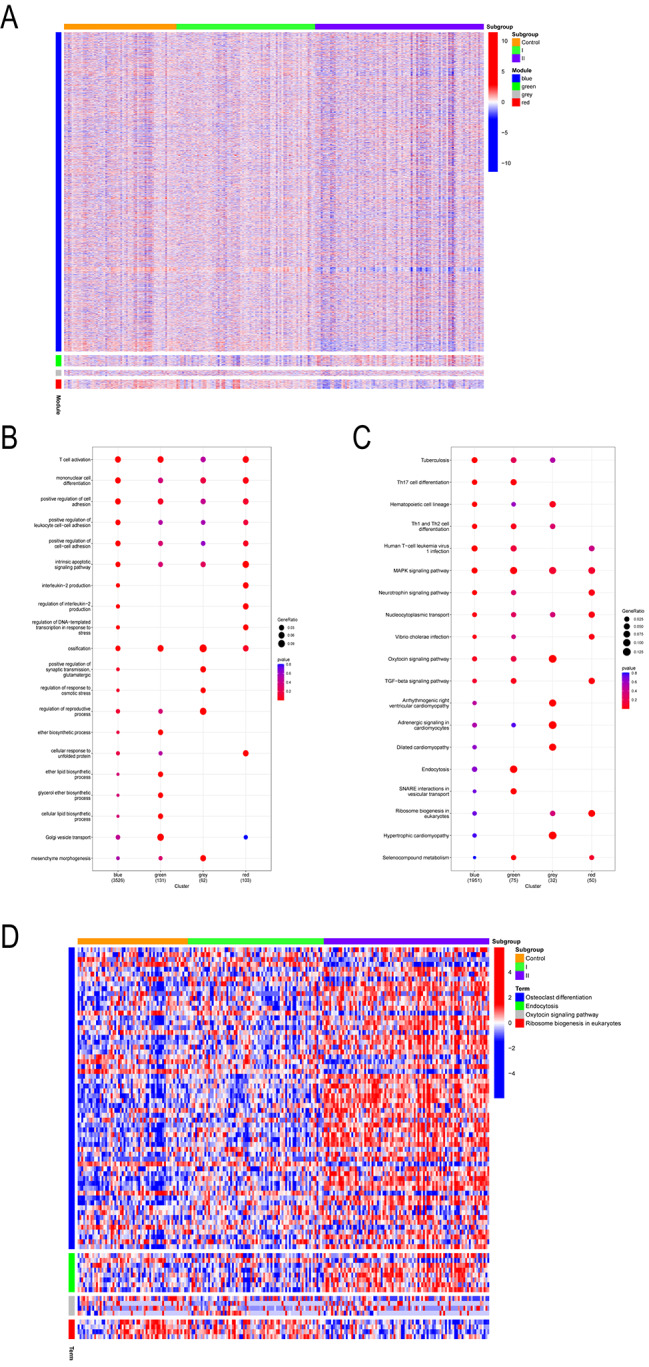
Correlation heatmap and functional enrichment analysis of two subgroups and four gene modules. A Correlation heatmap in different subgroups and modules. B GO enrichment analysis of top 20 biological processes. C Enrichment analysis of the top 20 KEGG pathways. D Correlation heatmap of different subgroups and KEGG significantly enrichment results

In the analysis of the correlation between different modules and gene subgroups, the results showed that in the subgroups II, the gene expression of blue and red module was lower, while that of green module was higher. On the contrary, in the subgroup I, the gene expression of green module was lower and that of red module was relatively higher (Fig. 8A). Besides, the results of ANOVA showed that both subgroup and hospital length of stay were independent predictors of OM severity (Tables 2 and 3, *P* < 0.05).

**Table 3:** Analysis of variance for subgroups and Hospital stays

	Degree of freedom	Sum square	Mean square	F value	*P* value
Subgroup	2	1180	590	5.187	0.0073**
Hospital stays	2	6679	3339	29.348	1.25e-10***
Residuals	94	10,696	114		

Abbreviations: *: *P* < 0.05; **: *P* < 0.01; ***: *P* < 0.001.

### GO and KEGG functional enrichment analysis

The biological processes and pathways of the four gene modules were concentrated and analyzed using the GO and KEGG databases. In GO analysis, the blue module genes were closely related to biological processes such as T cell activation, leukocyte adhesion and immune response. The green module genes were closely related to biological processes such as cellular lipid biosynthetic process, cellular response to antibiotic and endoplasmic reticulum to Golgi vesicle-mediated transport. The grey module genes were closely related to biological processes such as ossification, mesenchyme morphogenesis and regulation of oxidative phosphorylation. The grey module genes were closely related to biological processes such as interleukin-2 production, intrinsic apoptotic and chondrocyte development (Fig. 8B).

In the analysis of KEGG pathway, the blue module was significantly enriched in Th17 cell differentiation pathway, green module was significantly enriched in interaction in vesicular transport, grey module was significantly enriched in PI3K-Akt signaling pathway, and red module was significantly enriched in TGF-beta signaling pathway (Fig. 8C). To further investigate the enrichment of KEGG pathway in different subgroups, a visual KEGG pathway heatmap was drawn. Genes related to ribosome biogenesis in eukaryotes in vesicular transport pathway were lowly expressed in subgroup II. Interestingly, genes related to osteogenic differentiation and endocytosis pathways were highly expressed in subgroup II and relatively low in normal samples. While no significant differences were found in the expression of genes related to oxytocin signaling pathway (Fig. 8D) (Additional file 3).

## Discussion

*S. aureus* is the most common pathogen of OM [9]. Exploring the mechanism of occurrence and development of OM mediated by *S. aureus* is utmost critical for timely and accurate diagnosis and targeted treatment of OM patients. A recent OM-related bioinformatics study revealed that two key proteins, PH domain and leucine rich repeat protein phosphatase 2 (PHLPP2) and epidermal growth factor (EGF), are core nodes of OM-related differential genes that may affect the interaction of *S. aureus* with osteoblast interactions [15]. However, the specific molecular pathophysiological mechanisms of OM remain unclear, making it difficult to derive definitive treatment options. OM has considerable clinical heterogeneity and the huge differences in patient groups have led to more than 10 typing methods [25]. Most classifications are related to the clinical symptoms and signs of patients, and there are no criteria for typing at the molecular level. Moreover, most of the previous studies focused on the differential gene analysis of OM cases and control cases [26–28], and there were no molecular subtypes based on transcriptome data.

To the best of our knowledge, we conducted the first study to classify patients with *S. aureus*-induced OM into multiple molecular subgroups by bioinformatics analysis, aiming to explore the association of each subgroup with clinical features and molecular mechanisms. In this study, we integrated samples from three independent GEO gene datasets, including healthy samples and OM samples. First, after eliminating the batch effect due to platform and batch, 164 OM patients were classified into two different subgroups using an unsupervised algorithm. Next, genes from OM patients were modularized by combining GSEA and WGCNA to further explore the associations between the clinical characteristics and gene modules. Finally, the biological functions and pathways of genes in each subgroup were further explored through GO and KEGG enrichment analyses to provide support for clinical severity grading of OM based on transcriptomics.

Previous studies have shown that molecular subgroup analysis of cancer can associate subgroups with internal and external indicators to predict the prognosis of subgroups and guide treatment. Zhang et al. [29] classified gastric adenocarcinoma into five subgroups based on single-cell sequencing data, three of which were highly compatible with pathologic features and one of which predicted poor prognosis in gastric adenocarcinoma. A recent large prospective study found that the subgroup of HER2-low-positive tumours survived longer than the HER2-zero subgroup after receiving neoadjuvant combination chemotherapy and may serve as a novel molecular subgroup for the clinical treatment of breast cancer [30]. Additionally, molecular subgroups are also widely used in the study of many non-tumor diseases such as coronary artery disease [31], chronic obstructive pulmonary disease [32] and idiopathic pulmonary fibrosis [33] to provide assistance for precision diagnosis and treatment. We divided the patients with OM into two subgroups and found that although there was no significant difference in age and sex between the two subgroups, the number of MRSA, the number of severe cases in subgroup II were higher than those in subgroup I, and the length of hospital stay in subgroup II was much longer than that in subgroup I, suggesting that patients in subgroup II might be more serious than subgroup I. Therefore, we can distinguish the above two subgroups according to different clinical characteristics, and further explore the molecular mechanism of the subgroups to guide the diagnosis and treatment of *S. aureus*-induced OM.

It is well known that the pathophysiological development of OM mediated by *S. aureus* involves the imbalance of differentiation ability and quantity of osteoblasts and osteoclasts, which is a bone destruction process in which the differentiation ability of osteoclasts is stronger than that of osteoblasts [34]. Several studies have demonstrated the fundamental role of osteoclast differentiation in *S. aureus*-induced OM. *S. aureus* enhances osteoclast differentiation in vitro, leading to bone loss. Furthermore, S. aureus induced differentiation of mouse RAW264.7 cells to osteoclasts, a process associated with increased NF-κB p65 phosphorylation as well as the nuclear factor of activated T cells c1 (NFATc1) expression [35]. It has also been found that *S. aureus* protein A (SPA), the main component of *S. aureus*, stimulates osteoclast differentiation through binding to IgG, accompanied by increased expression of the osteoclast differentiation-related gene NFATc1 [36]. In our study, the pathway of osteoclast differentiation was significantly enriched in subgroup II. Most notably, the ability of genes related to osteoclast differentiation was enhanced in the pathological process of OM [37], which is consistent with the rich results of genes related to osteoclast differentiation in subgroup II and may indicate that the pathway of osteoclast differentiation is associated with more severe molecular subtypes of OM.

KEGG enrichment results revealed that there were 62 main targets involved in osteoclast differentiation, and the first three main targets included cathepsin K (CTSK), protein kinase B (AKT2) and B cell linker (BLNK). CTSK, an acidic cysteine endonuclease produced by osteoclasts, has been shown to play a role in bone resorption under inflammatory conditions [38]. One study found significantly higher CTSK expression in infected OM samples compared to normal bone tissue, which may lead to lysis of bone tissue [39]. In recent years, CTSK has been widely used as a biomarker of osteoclast differentiation to study the mechanism of OM. The inflammatory factor interleukin-1 (IL-1) promoted RANKL-induced CTSK expression in osteoblasts and stimulated activation of the major transcription factor NFATc1 [40]. The inflammatory environment simulated by SPA can significantly promote the expression of CTSK in osteoclasts via the NF-κB pathway, and the expression of CTSK was inhibited by the addition of inhibitors of this pathway, which provided a potential target for the treatment of OM. Therefore, inhibition of CTSK expression may inhibit or even reverse osteoclast differentiation and slow the progression of bone destruction [41]. We may consider the use of CTSK-related inhibitors to improve the condition of patients with subgroup II OM. However, the exact efficacy needs to be validated by in-depth basic research and clinical trials.

In addition, the pathway of SNARE interactions in vesicular transport was enriched in subgroup II. Related studies showed that SNARE produced inflammatory mediators by reducing nuclear translocation of NF-κB [42]. Osteoblasts delivered synthetic bone matrix proteins to the bone surface, and SNAREs played a specific role in mediating nuclear fusion in this process [43]. Therefore, we hypothesize that the pathway of SNARE interactions in vesicular transport may play a role in osteoblast-associated protein transport under OM conditions. However, whether genes related to this pathway, such as ACTR2, can be used as targets of action requires further in-depth mechanistic studies.

Our study has several certain limitations. Firstly, despite good consistency was found for the two OM subgroups, validation is still needed in combination with more transcriptomic and large samples of clinical data. Secondly, although we incorporated transcriptomic data from three datasets, future integration of data from emerging omics such as metabolomics is needed to provide guidance on diagnostics and personalized new drug development for OM molecular subgroups.

## Conclusion

This study identified a novel molecular subgroup of OM caused by *S. aureus* based on transcriptomic data, and further analyzed the clinical features, immune microenvironment and biological functions of the DGEs among the subgroups. The upregulated genes related to the osteogenic differentiation pathway suggested that OM subgroup II is more severe. CTSK, the main target of this pathway, may be a potential therapeutic target for patients with OM subgroup II.

## Electronic supplementary material

Below is the link to the electronic supplementary material.

Supplementary Material 1

Supplementary Material 2

Supplementary Material 3

## Data Availability

The microarray data used to support the findings of this study can be downloaded from the GSE6269, GSE16129 and GSE30119 datasets (https://www.ncbi.nlm.nih.gov/geo). The processed data are available from the corresponding author upon request.

## Abbreviations

OM: Osteomyelitis
*S. aureus*: *Staphylococcus aureus*
GEO: Gene expression collection
DEGs: Differentially expressed genes
GO: Gene ontology
KEGG: Kyoto encyclopedia of genes and genomes
PPI: Protein–protein interaction
GSEA: Gene set enrichment analysis
ES: Enrichment score
FDR: False discovery rate
CDF: Cumulative distribution function
PCA: Principal component analysis
WGCNA: Weighted gene co-expression analysis
TOM: Topological overlap matrix
CIBERSORT: Cell Type Identification by Estimating Relative Subsets of RNA Transcripts
PHLPP2: PH domain and leucine rich repeat protein phosphatase 2
EGF: Epidermal growth factor
NFATc1: Nuclear factor of activated T cells c1
SPA: *S. aureus* protein A
CTSK: Cathepsin K
AKT2: Protein kinase B
BLNK: B cell linker. IL-1:interleukin-1
