# Supplementary material for: Identification of molecular subgroups in osteomyelitis induced by staphylococcus aureus infection through gene expression profiles
Source: BMC Med Genomics. 2023 Jun 27;16:149. doi: 10.1186/s12920-023-01568-x (PMC10304621; doi:10.1186/s12920-023-01568-x)
Supplement: Supplementary file 1 — Supplementary Material 1 [file 12920_2023_1568_MOESM1_ESM.pdf]

Supplementary Table S1. The number of DEGs by case-control and case-case comparisons.

Subgroups	The specific DEGs were compared with the normal group	The specific DEGs were compared with another subgroup	The specific up-regulated DEGs were compared with another subgroup
Subgroup I	1049	4049	2172
Subgroup II	4954	4049	1877
Total	6003	8089	4049
