# Supplementary material for: Identification of molecular subgroups in osteomyelitis induced by staphylococcus aureus infection through gene expression profiles
Source: BMC Med Genomics. 2023 Jun 27;16:149. doi: 10.1186/s12920-023-01568-x (PMC10304621; doi:10.1186/s12920-023-01568-x)
Supplement: Supplementary file 2 — Supplementary Material 2 [file 12920_2023_1568_MOESM2_ESM.pdf]

Supplementary Table S2. The number of DEGs by case-control and case-case comparisons.

Gene modules	The specific up-regulated DEGs
Blue	AAAS/AAK1/AARS/AATK/ABCA1/ABCA5/ABCA7/ABCB1/ABCB4/ABCB6/ABCB7/ABCC1/ABCC2/ABCC5/ABCD4/ABCE1/ABCF2/ABCG1/ABHD11/ABHD2/ABHD3/ABHD4/ABHD5/ABHD8/ABI1/ABL2/ABLIM1/ABR/ABT1/ACAA1/ACACB/ACADM/ACADSB/ACOT9/ACOX1/ACOX2/ACP1/ACP2/ACP6/ACPP/ACSBG2/ACSL1/ACSL4/ACSM3/ACTA2/ACTB/ACTN1/ACTN4/ACTR1A
Green	ACTR2/ACVR2A/ADAM10/ARFGEF1/ARHGAP19/ARPC5/ATP11B/ATP6V1C1/ATXN3/AZI2/B9D2/BAZ1A/BAZ2B/BICD2/C1GALT1C1/CAB39/CACNA1I/CAP1/CAPZA1/CBX1/CCDC6/CD300A/CDC42EP2/CIRBP/CLCC1/CLDN17/CLTC/CNIH4/CTBP2/CUL4B/DHRS7B/DICER1/DR1/EDEM3/EMP3/EVI2A/FASN/FBXW2/FNBP1L/FRAT1/FXYD5/GGA2/GLRX/GNPAT/GPSM2/GUCY2D/HADHB/HERC4/HIGD2A
Red	A4GNT/ANP32D/ANXA10/ASPN/BTG4/C8A/CACNB4/CACNG2/CHRNG/DDX25/DKFZP434L187/EDN2/EPB41L4A/EPX/FAM131B/FCN3/FLT4/FOXC2/GABRB3/GFRA4/GJA8/GPR37L1/GUCA2A/HNF4G/HS3ST3B1/IFNA5/IL5/ITGA1/KCNJ6/KCNQ2/MGAT4C/MGP/MLC1/MSH4/MUC16/MYH8/NPPC/NRXN1/NUPR1/NXF3/PAGE4/PCK1/PIR/PLK1/PPP1R1A/PRKAA2/RAPGEF4/RAX/RIPK4
Grey	ADNP2/AP3M2/ARHGAP12/ATP6V1D/AUH/AVPI1/BAG3/BCLAF1/BRD1/BRD3/BTG3/BZW1/CBLL1/CCDC59/CCT6B/CD83/CHD1/CLCF1/CLEC2B/COPS8/CRY1/CTH/DBF4/DCP1A/DCTN6/DDX21/DNTTIP2/DUSP12/DUSP2/EFNA4/EGR2/EIF2AK3/ELF1/EPB41L5/FKBPL/FXR2/G3BP2/GMEB2/GNE/GTPBP4/HDGFRP3/HERPUD1/HIC2/ICOSLG/ID2/IKZF5/KEAP1/KIAA0040/KLF9

Abbreviations: DEGs: differentially expressed genes.
