# Supplementary material for: Identification of molecular subgroups in osteomyelitis induced by staphylococcus aureus infection through gene expression profiles
Source: BMC Med Genomics. 2023 Jun 27;16:149. doi: 10.1186/s12920-023-01568-x (PMC10304621; doi:10.1186/s12920-023-01568-x)
Supplement: Supplementary file 3 — Supplementary Material 3 [file 12920_2023_1568_MOESM3_ESM.pdf]

Supplementary Table S3. Significant enrichment analysis of KEGG pathway (top 1).

Gene modules	Description	*P* value	Gene ID	Number
Blue	Osteoclast differentiation	1.054410188959 67e-09	AKT2/BLNK/CAMK4/CHUK/CTSK/CYBA/CYLD/FCGR1A/FCGR2A/FCGR2C/FCGR3B/FOS/FOSL2/FYN/GAB2/GRB2/IFNAR1/IFNG/IFNGR1/IFNGR2/IL1B/IL1R1/IRF9/JAK1/JUNB/LCK/LILRA2/LILRA3/LILRA5/LILRA6/LILRB2/LILRB3/LILRB4/MAP2K6/MAPK1/MAPK10/MAPK14/MAPK3/NCF1/NCF2/NCF4/NFATC1/PIK3CB/PPARG/PPP3CC/PPP3R1/RAC1/RELB/SIRPA/SIRPB1/SIRPG/SOCS3/SPI1/STAT1/STAT2/SYK/TGFBR1/TGFBR2/TNF/TNFRSF1A/TRAF2/TYROBP	62
Green	Endocytosis	0.00204268758041 42	ACTR2/SMAD3/ARFGEF1/ARPC5/CAPZA1/CLTC/STAM2/ZFYVE16	8
Red	Ribosome biogenesis in eukaryotes	0.00433281969213 434	GTPBP4/NMD3/NXT1/RCL1	4
Grey	Oxytocin signaling pathway	0.00291115328633 161	CACNB4/CACNG2/KCNJ6/PRKAA2	4
